# Motor-enriched learning for improving pre-reading and word recognition skills in preschool children aged 5–6 years – study protocol for the PLAYMORE randomized controlled trial

**DOI:** 10.1186/s12887-020-02430-0

**Published:** 2021-01-04

**Authors:** Anne Kær Gejl, Anne Sofie Bøgh Malling, Linn Damsgaard, Anne-Mette Veber-Nielsen, Jacob Wienecke

**Affiliations:** 1 Integrative Physiology, Department of Nutrition, Exercise and Sports, University of Copenhagen, Nørre allé 51, 2200 Copenhagen, Denmark; 2 National Centre for Reading, University College Copenhagen, Humletorvet 3, 1799 Copenhagen, Denmark

**Keywords:** Motor-enriched learning, Embodied learning, Physically active lessons, Pre-reading skills, Word recognition, Preschool children

## Abstract

**Background:**

Results from previous studies suggest that bodily movements, spanning from gestures to whole-body movements, integrated into academic lessons may benefit academic learning. However, only few studies have investigated the effects of movement integrated into reading practice. The PLAYMORE study aims to investigate the effects of two interventions focusing on a close and meaningful coupling between bodily movement and academic content on early pre-reading and word recognition skills in children. Further, the study aims to compare two interventions involving either hand movements (i.e. using arms and hands) or whole-body movements (i.e. using the whole body). Potential mediating factors underlying the link between bodily movement on early pre-reading and word recognition skills will be explored.

**Methods/design:**

The PLAYMORE study will be conducted as a three-armed randomized controlled trial including children aged five to six years recruited from four schools in the Copenhagen area, Denmark. Stratified by class, children will be randomly allocated to one of three 8-week intervention/control periods: 1) teaching involving whole-body movements, 2) teaching involving hand movements (i.e. arms and hands) or 3) teaching involving minimal motor movements (i.e. seated on a chair using paper and pencil). Outcome measurements, including pre-reading and word recognition skills, will be collected before and after the intervention period to assess the intervention effects. This study protocol follows the SPIRIT guidelines.

**Discussion:**

The PLAYMORE study will add to the current knowledge concerning the link between bodily movement and academic performance with important details about pre-reading and word recognition skills in preschool children. If effective, evaluation of the implementation of the PLAYMORE program should be conducted in order to investigate whether the effects can be transferred into standard school settings. The PLAYMORE study will lay the foundation for future research that have the potential to inform the political and scientific debate and importantly, to provide teachers with detailed information of how to implement movements effectively during teaching in order to support and motivate children in the process of learning to read.

**Trial registration:**

The study was retrospectively registered in ClinicalTrials.gov (NCT04618822) the 5th of November 2020.

## Background

The acquisition and development of competent reading skills in childhood are critical to functioning and well-being later in life. Poor spelling and reading skills in children and adolescents have been associated with poor academic achievement [67, 74, 89], school dropout [20, 55], and lower occupational status in adulthood [62, 74]. Initially, when learning to read, the acquisition of phoneme awareness and letter knowledge is of great importance. Letter knowledge is required in order to understand the alphabetic code, that is, the fact that sounds and letters can combine, which, in turn, will allow for the development of basic word reading accuracy. Accordingly, phoneme awareness and letter knowledge measured before the outset of formal reading instruction are unique predictors of later reading and spelling abilities [11, 29, 36, 48, 56, 68]. Given the fundamental role of reading abilities in modern society, it is essential to identify forms of instruction and practice that support and motivate children in the process of learning to read with a specific focus on the acquisition of phoneme awareness, letter knowledge and decoding abilities in the first school year.

School-based interventions involving bodily movement (spanning from less pronounced movements such as e.g. gestures to physical activity (PA) in terms of e.g. exercise) have been recognized as a potential strategy to support academic learning in children and adolescents and several reviews in this field have been published (e.g. [3, 18, 22, 23, 51, 58–60, 71, 87]). In general, the effects on overall academic performance are found to be small, also when summarizing isolated effects within subdomains such as reading and mathematics [3, 22, 23, 51, 71]. However, bodily movement is a multifaceted construct and the mechanisms through which cognition and learning are effected depend highly on the characteristics of the movement activities (e.g. type, movement range) [5, 19, 53]. Thus, when investigating the link between bodily movement and academic performance, it is important to differentiate between different types of movement activities. During the last decade, much research has focused on the relationship between general PA, in terms of e.g. physical exercise, cognitive performance and academic learning (for reviews see e.g. [24, 71]). From laboratory-based and strictly controlled studies, it is evident that cognitive performance, in particular executive functions (which play an important role in the development of e.g. word recognition and spelling skills [12, 39, 80]) can be enhanced following a single bout of PA ([15, 34, 44], for review see [14, 35]). Moreover, though equivocal, results from some experimental studies suggest that several bouts may benefit executive functions in children and adolescents [21, 33, 79]. Physiological mechanisms underlying the acute effects of PA on cognition comprise transient increase in peripheral levels of catecholamines [16, 72], steroid hormones [8, 32] and growth factors [28, 72] as well as changes in cerebral oxygenation and cerebral blood flow [25]. In addition, increased catecholamines and growth factor levels induced by several bouts of PA can eventually lead to e.g. structural brain alterations (i.e. new vascular and neural structure) affecting cognitive performance (for review see [81]). However, whether this effect transforms into improved academic learning, when PA is conducted in a school setting, is still unclear, since high quality school-based intervention studies are limited and often conducted as multi-component studies including PA as one of more intervention components.

Another promising, but less investigated, approach is the coupling of movement and academic content during academic lessons. This approach rely on principles drawn from theories of embodied cognition and is often referred to as *embodied learning* [49, 73]. Briefly, embodied cognition suggests that human cognition develop from the body’s interactions with the surrounding environment [70]. Interestingly, it has been demonstrated that both visual and motor brain networks are activated during cognitive tasks such as reading and problem-solving, suggesting that task performance not only rely on e.g. visual information but also sensory information gained through bodily experiences (discussed by e.g. [90]). As such, actions involving task relevant information may support learning of the specific task in addition to just hearing or observing it (i.e. the enactment effect) [26]. Based on this view, embodied learning activities aim to create a close coupling between the movement and the specific task to be acquired, but vary in regards to e.g. the level of bodily engagement, task integration [73] and congruency between the movement and the task at hand [40]. Over the years, a large body of research has emerged mainly using gestures or hand movements but also whole-body movements during learning (e.g. [2, 5, 19, 53, 66]). For example, Mavilidi and colleagues used gestures and physical exercise during teaching of 3–5 year-old children learning a new language over a 4-week period and evaluated the effects on new words learned [53]. Using a four-armed randomized controlled design, they found that gestures as well as physical exercise, particularly when movements were integrated into the learning task, had beneficial effects on learning. A recent systematic review (*unpublished, in review*) summarized studies investigating the effects of bodily movement on early first language word recognition and spelling skills in children and found that only few studies of limited quality focused on interventions founded in the embodied learning theory, i.e. involving activities with a close coupling between the movement and the academic task. The identified studies mainly focused on handwriting [17, 46, 61, 69, 84, 85], copying or tracing letters/words [6, 37, 46, 50, 85, 91] and only four studies included whole-body movements (i.e. embodying letters, walking the outline of letter) [4, 7, 19, 52]. Considering the limited amount of studies involving whole-body movements in combinations with the large variety of movement components included in these studies, it is still uncertain whether such activities have a larger impact on word recognition and spelling skills compared to teaching methods not incorporating motor activities. Such information is needed in order to enable a construction of general guidelines and specific instructions for integrating PA in a meaningful way into the school day.

Consequently, the current study aims to investigate the effects of two interventions focusing on movement activities with a close and meaningful coupling between the movement and the academic content on pre-reading and word recognition skills in children. Previous studies focusing on mathematics [5] or second language [53] suggest that the effects may depend on the motor modality used (i.e. whole-body or hand movements). Thus, another aim of the study is to compare the two interventions involving either hand movements (i.e. using arms and hands) or whole-body movements (i.e. using the whole-body). Lastly, to explore potential mediating factors underlying this relationship, the intervention effects on intrinsic motivation and cognitive performance will be evaluated. The study will be conducted as a three-armed randomized controlled trial designed to test superiority of two interventions as compared with a control. Included subjects will be randomly allocated in an approximate 1:1:2 fashion to either 1) teaching involving whole-body movements, 2) teaching involving hand movements or 3) teaching involving minimal motor movements (i.e. seated on a chair using paper and pencil).

It is hypothesized that the two interventions involving hand or whole-body movements, respectively, will induce a larger effect on pre-reading and word recognition skills compared to a teaching method where movement is reduced to a minimum (i.e. seated using paper and pencil). In addition, based on current literature within mathematics [5], it is expected that the intervention involving whole-body movements induces the largest effects compared to the intervention involving hand movements.

## Methods/design

The study was retrospectively registered in ClinicalTrials.gov (NCT04618822) the 5th of November 2020. This study protocol (1st version, October 2020) follows the SPIRIT guidelines [13].

### Study design

**Fig. 1:**
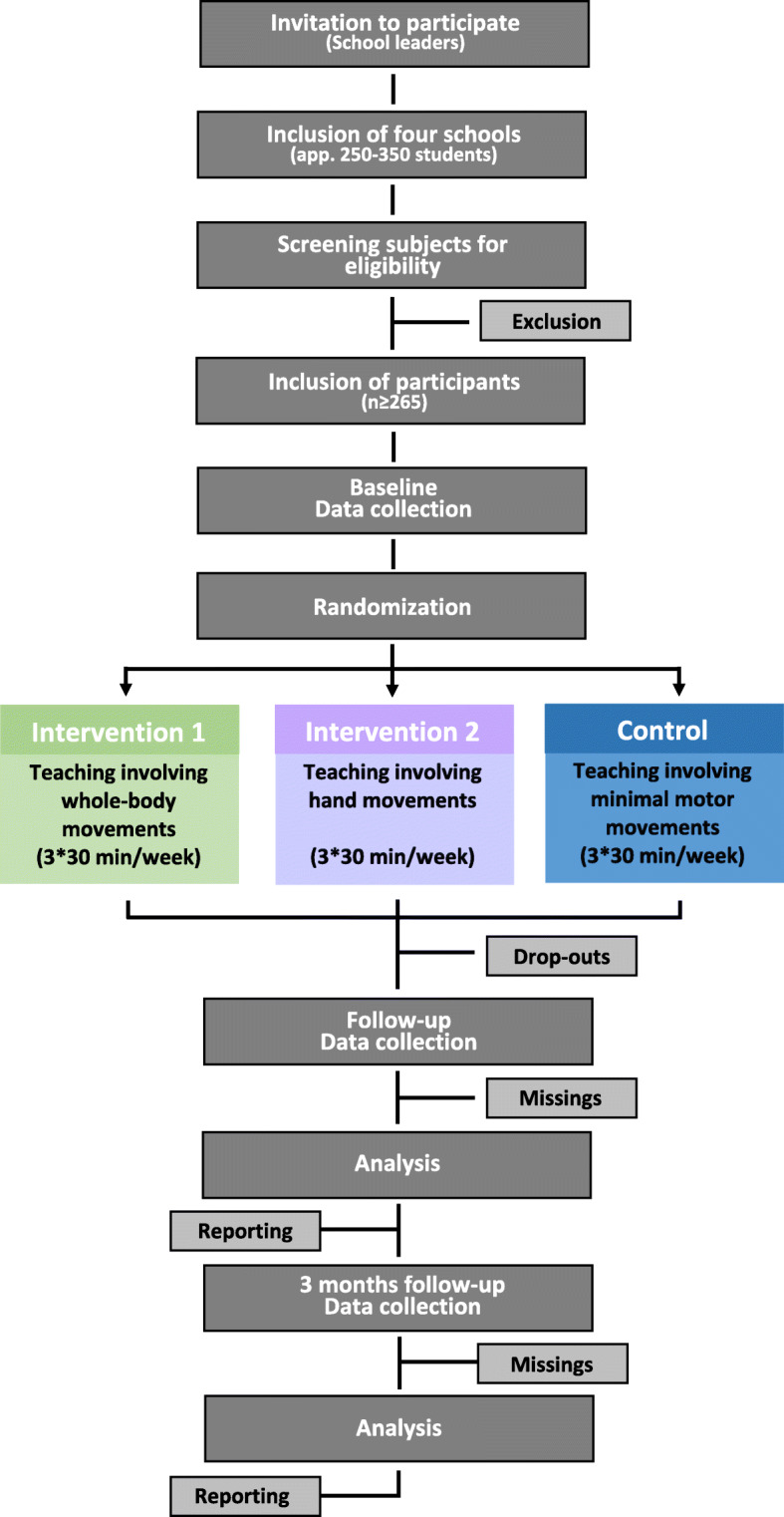
Study design and flow of participants

The study will be conducted as a three-armed randomized controlled trial including two intervention groups and one control group (CG) (Fig. 1, study design and flow of participants). Intervention activities will be completed as either whole-body movements (WM, intervention group I) or hand movements (HM, intervention group II) over an 8-week period at four primary schools. Using random selection of sealed envelopes, participants will be randomized stratified by class in an approximate 1:1:2 fashion to either WM, HM or CG. Specifically, within each class six subjects will be assigned to WM, six subjects to HM, while the remaining subjects will constitute the CG. The project leader will be responsible for all steps related to the enrollment and allocation of participants. Outcome measurements will be collected before and after the intervention period enabling assessment of the intervention effects. A schematic diagram of time schedule of enrolment, interventions and assessments are presented in Table 1.

**Table 1:** Schedule of enrolment, interventions and assessments in the PLAYMORE study

		STUDY PERIOD											
Activity or assessment	School/class /group /individual level	Recruite-ment	Baseline	Intervention period (8 weeks)								Post	3-month follow-up
				1	2	3	4	5	6	7	8		
Contact to schools	School	**●**											
Eligibility screen	Individual	**●**											
Consent (parents)	Individual	**●**											
Inclusion	Individual	**●**											
Randomization	Individual		**●**										
**INTERVENTIONS/CONTROL** (3*30 minutes/week)													
Whole-body movements	Group (6 subjects)			**●●●**	**●●●**	**●●●**	**●●●**	**●●●**	**●●●**	**●●●**	**●●●**		
Part-body movements	Group (6 subjects)			**●●●**	**●●●**	**●●●**	**●●●**	**●●●**	**●●●**	**●●●**	**●●●**		
Control	Group (app. 12 subjects)			**●●●**	**●●●**	**●●●**	**●●●**	**●●●**	**●●●**	**●●●**	**●●●**		
**ASESSMENTS**													
***Reading skills***													
Word reading with pictures	Individual		**●**									**●**	**●**
Word reading without pictures	Individual		**●**									**●**	**●**
Naming of letter sounds	Individual		**●**									**●**	**●**
Letter Fluency	Individual		**●**									**●**	**●**
Letter test 2	½ class		**●**									**●**	**●**
Word reading test 1	½ class		**●**									**●**	**●**
Knowledge of trained letter- sound correspondences	½ class		**●**									**●**	**●**
***Cognitive performance***													
Digit Span	Individual		**●**									**●**	**●**
N-back	Individual		**●**									**●**	**●**
***Motor skills***													
Flamingo Balance test	½ class		**●**									**●**	**●**
9-hole pegboard	Individual		**●**									**●**	**●**
***Compliance data***													
Presence	Individual			**●●●**	**●●●**	**●●●**	**●●●**	**●●●**	**●●●**	**●●●**	**●●●**		
Active participation	Individual			**●●●**	**●●●**	**●●●**	**●●●**	**●●●**	**●●●**	**●●●**	**●●●**		
Session completion	Group			**●●●**	**●●●**	**●●●**	**●●●**	**●●●**	**●●●**	**●●●**	**●●●**		
Compliance to protocol	Group			**●●●**	**●●●**	**●●●**	**●●●**	**●●●**	**●●●**	**●●●**	**●●●**		
***Intrinsic motivation***	Individual				**●**		**●**		**●**		**●**		

Adapted from the SPIRIT guideline (Chan et al., 2013)

### Participants and recruitment

The intervention is targeted children five to six years old who have just started school. In Denmark, children start school in August of the calendar year in which they turn six. However, formal reading instruction is not a part of the first school year (referred to as “*Grade 0*”) which has been compulsory in Denmark since 2009 but remains a preparation year [78]. Thus, teachers are required to prepare students for formal reading instruction in later grades and letter knowledge is emphasized as an area meriting special attention [78]. The intervention period will start approximately 1.5 months after children have started Grade 0. At the beginning of Grade 0, students are usually able to identify at least half of the letters of the alphabet [41, 65], but only a few students will be able to read words [65].

School leaders of primary schools in the Copenhagen area will be contacted by phone and invited to an initial non-committal meeting. All preschool teachers at the respective schools are encouraged to participate in this meeting. If the school leader and teachers agree to participate in the study, logistic planning will be done individually with all teachers to ensure that the final time schedule is in agreement with the respective teacher and class. All preschool children from schools enrolled in the study will be invited to participate in the project activities and assessments, however, children who do not speak any Danish or children with cognitive or physical disabilities hindering participation in the project activities will be excluded from the randomization and the final dataset and analyses. These children will be invited to attend the control activities. Further, only children whose parent(s) or legal guardian(s) provide signed written informed consent will complete the assessments. Children without consent will attend the control activities in the classroom but will not be included in the assessments and thus not the final dataset or analyses. All children will follow the interventions/control that they were initially allocated to.

### Structure and learning content of the activities

During the 8-week intervention period, three sessions of approximately 30 min duration will be completed each week, counting 24 sessions in total. At the schools, trained instructors will administer activities in the two intervention groups, while the usual schoolteachers will administer the control activities. Activities focus on the acquisition of letterforms, letter-sound correspondences and reading and spelling of short words. The learning content of the activities is identical in the three groups and vary mainly in regard to the degree of bodily movement. Activities are developed based on the research-founded Danish teaching material, *Fandango Mini*, which is recognized and used by several preschool teachers in Denmark [38]. The material is based on a synthetic phonics approach and is scheduled as a 20-week systematic course covering both standard and conditional pronunciations of the letters. From the very beginning, students practice reading and spelling of words composed of the letter-sounds trained so far.

The first six weeks follow the same weekly structure involving the same type of activities. During each of these six weeks, four to five new letters and related sounds and two to four new words containing these letters and sounds (*target words*) will be studied intensively (Table 2). Additional words not classified as target words will be presented to the children during the intervention period, but focus will be on studying the target words. The last two weeks, activities link what has been learned during the first six weeks and involve activities focusing on word reading and the composition of minor sentences containing the words trained. Thereby, the children will be taught 25 letters and the related sounds (standard and conditional pronunciations) and 18 target words during the intervention period. An example of a protocol for one week (week 1–6) and detailed description of intervention−/control activities are provided in Table 3. Components specific for each intervention- or control condition are described in the sections below.

**Table 2:** Letters, letter sounds, and target words trained during each week of the intervention period

Week	1							2					3					4					5					6						7 and 8
**Letters**	l	å	s	m	e			b	i	u		y	n	k	f	a		æ	h	t	d		g	p	o		j	ø	r		c	v		all
**Letter sounds**	[l]	[ɔ]	[s]	[m]	[e]	[ɛ]	[ə]	[b]	[i]	[u]	[ɔ]	[y]	[n]	[k]	[f]	[a]	[ɑ]	[ɛ]	[h]	[t]	[d]	[ð]	[g]	[p]	[o]	[ʌ]	[j]	[ø]	[ʁ]	[ɐ̯]	[s]	[v]	[w]	all
**Target words [pronunciations]**	lås [ˈlɔˀs]^a^							bus [ˈbus]					fly [ˈflyˀ]					dæk [ˈdεg]					gås [ˈgɔˀs]					sø [ˈsøˀ]						båd [ˈbɔˀð]
	mel [ˈmeˀl]							bil [ˈbiˀl]					nul [ˈnɔl]					hat [ˈhad]					kop [ˈkʌb]					rive [ˈʁiːvə]						mur [ˈmuɐ̯ˀ]
																		hest [ˈhεsd]					majs [ˈmɑjˀs]					hav [ˈhɑw]						
																							kano [ˈkæːno]											

^a^The Danish prosodic feature *stød* (a type of creaky voice [31]) is denoted withˀ

**Table 3:** Description of intervention−/control activities

PROTOCOL 1 WEEK (EXAMPLE: WEEK 1) Letters in focus: S, E, L, M, Å Story: River Regatta				
**SESSION I**				
**Activity**	**Focus**	**WM**	**HM**	**CG**
**Introduction** (5 min)		While children are standing in acircle, the instructor introducesherself/himself and the interventionbriefly. Children’s names must beremembered.	While children are sitting around thetable, the instructor introducesherself/himself and the interventionbriefly. Children’s names must beremembered.	While the children are sitting at theirtable, the teacher introduces thecontrol intervention briefly.
**Story telling** (5 min)	Presentationofthe weeklystory abouttheflying suitcase	*”The next 8 weeks we are going toplay a lot with letters. I know, thatyou’ve already learned some lettersand that you are already very good atletters. But listen … this morning …*”➔ a story about the flying suitcaseon river regatta is read aloud. Materials: Letter written by the flying suitcase Suitcase	*”The next 8 weeks we are going toplay a lot with letters. I know, thatyou’ve already learned some lettersand that you are already very good atletters. But listen … this morning …”*➔ a story about the flying suitcaseon river regatta is read aloud. Materials: Letter written by the flying suitcase Suitcase	*”The next 8weeks we are going to playa lot with letters. I know, that you’vealready learned some letters and thatyou are already very good at letters.But listen … this morning …”* ➔ astory about the flying suitcase onriver regatta is read aloud. Materials: Letter written by the flying suitcase Suitcase
**Activity I** (2 min) Title:” *Letterrhyme*” Letter: S	Presentationofletter sound	In continuation of the story, a letterrhyme with S/[s] is read aloud. Aletter rhyme consists of words ofwhich the majority begins with thesound in focus – in this case S/[s].	In continuation of the story, a letterrhyme with S/[s] is read aloud. Aletter rhyme consists of words ofwhich the majority begins with thesound in focus – in this case S/[s].	In continuation of the story, a letterrhyme with S/[s] is read aloud. Aletter rhyme consists of words ofwhich the majority begins with thesound in focus – in this case S/[s].
**Activity II** (3 min) Title:” *Lettersound*” Letter: S	Presentationand practice ofletter sound-movementcoupling	While standing in a circle (markedwith six blackboard cloths), themovement-sound coupling,” *bodyphoneme*”, for the letter sound [s] isintroduced. The movement is practiced collectively while movingaround in a circle in the readingdirection. Materials: Six blackborad cloths placed in a circle	While sitting around the table, themovement-sound coupling,” *handphoneme*”, for the letter sound [s] isintroduced. The movement is practiced collectively in the readingdirection.	(not part of control session)
**Activity III** (2 min) Title:” *Symbol-soundcoupling*” Letter: S	Couplingbetween lettersound andletter form	The letter sound is here coupled tothe letter form. Children are askedwhat letter that is pronounced [s].When the letters S is identified,children are asked to draw threelarge S-letters on a large blackboardcloth attached to the floor. When thethree letters have been drawn, children are asked to trace the letterform until all children have completed the task. Materials: Six blackboard cloths placed in a circle Six pieces of chalk	The letter sound is here coupled tothe letter form. Children are askedwhat letter that is pronounced [s].When the letters S is identified,children are asked to draw three S-letters on a small blackboard. Whenthe three letters have been drawn,children are asked to trace the letterform until all children have completed the task. Materials: Six small blackboards, one for each child Six pieces of chalk	The letter sound is coupled to theletter form. Children are asked whatletter that is pronounced [s]. Whenthe letters S is identified, children areasked to write S-letters on a sheet ofpaper with guiding lines. Materials: Control sheet
**Activity IV** (5 min) Title: “*Soundhunt*” Letter: S	Identification ofletter sound	Children are told to stand behind thethree large S-letters. Then words inwhich the [s] sound is in the beginning, in the middle or in the end ofthe word are said aloud by the instructor. Children are then asked toidentify the location of the sound (i.e.beginning, middle or end) and jumpto the one of the S-letters written onthe blackboard cloth and performthe body phoneme while saying thesound. Beginning: jump to the rightS-letter, middle: jump to the middleS-letter, end: jump to left S-letter Materials: Words with [s] in the beginning,middle or end. Six blackboard clothsplaced in a circle.	Children are told to place theblackboard with the three S-letters infrom of them. Then words in whichthe [s] sound is in the beginning, inthe middle or in the end of the wordare said aloud by the instructor. Children are then asked to identify thelocation of the sound (i.e. beginning,middle or end) and place a smallblock on one of the S-letters writtenon the blackboard and perform thehand phoneme while saying thesound. Beginning: place the brick onthe right S-letter, middle: place thebrick on the middle S-letter, end:place the brick on the left S-letter Materials: Words with [s] in the beginning,middle or end. Six small blackboardsand bricks.	Children have a task on the controlsheet with pictures representingwords in which the [s] sound is inthe beginning, in the middle or inthe end of the word. Below eachpicture are three boxes. Children areasked to identify the location of thesound (i.e. beginning, middle or end)and mark the correct box. Beginning: the right box, middle: themiddle box, end: the left box Materials: Control sheet
**Story telling** (1 min)		The story about the flying suitcase onriver regatta is continued.	The story about the flying suitcaseon river regatta is continued.	The story about the flying suitcase onriver regatta is continued.
**Activity I-III** (7 min) Letter: E	See activity I-III	In continuation of the story, a letterrhyme with E/[e] (simple sound) isread aloud and the [e] “*bodyphoneme*” are presented andpracticed. Children are asked to writethree large E-letters on the blackboard cloth (see description of activity I-III). “*Sound hunt*” is onlycompleted for the first letter sound(in this case S). Materials: See Activity I-III	In continuation of the story, a letterrhyme with E/[e] (simple sound) isread aloud and the [e] “*handphoneme*” is presented and practiced.Children are asked to write threesmall E-letters on the blackboard (seedescription of activity I-III). “*Soundhunt*” is only completed for the firstletter sound (in this case S). Materials: See Activity I-III	In continuation of the story, a letterrhyme with E/[e] is read aloud.Children are asked to write E-letterson a sheet of paper with guidinglines. “*Sound hunt*” is only completedfor the first letter sound (in this case S). Materials: Control sheet
**Activity V** (6 min) Title: “*Soundduel*” Letter: E	Identification of simple and conditional sounds	The letter e has more than onesound. Two more pronunciations(conditional sounds, [ɛ] and [ə]) andrelated “*body phonemes*” arepresented. When the “*bodyphonemes*” have been practiced (seeactivity II), children are told to standwith their backs to each other inpairs of two. Then words includingone of the three e-pronunciations(simple or conditional) are readaloud. Children are instructed toidentify the correct sound and related “*body phoneme*” by themselves.Then the instructor counts downfrom three and the two childrenmust face each other while sayingthe sound and performing the related “*body phoneme*”. If incorrect, thechildren are told to discuss which letter sound is the correct. Correctionsare made collectively.	The letter e has more than onesound. Two more pronunciations(conditional sounds [ɛ] and [ə]) andrelated “*hand phonemes*” arepresented and practiced (see activityII). In pairs of two, children are thentold to sit facing each other withtheir hand covering their eyes. Thenwords including one of the three e-pronunciations (simple or conditional) are read aloud. Children areinstructed to identify the correctsound and related “*hand phoneme*”by themselves. Then the instructorcounts down from three and the twochildren must remove their handsand face each other while saying thesound and performing the related“*hand phoneme*”. If incorrect, the children are told to discuss which lettersound is the correct. Corrections aremade collectively.	The letter e has more than onesound. Two more pronunciations(conditional sounds) are presented. Children have a task on the controlsheet with three encircled pictures inthe center – each picture illustrate aword containing one of the three e-pronunciations. Around the encircledpictures are task-pictures illustratingother words containing one of thethree e-pronunciations. Children aretold to draw lines from each of thetask picture to the encircled picturecontaining the same e-pronunciation.Corrections are made collectively. Materials: Control sheet
**SESSION II**				
**Activity**	**Focus**	**WM**	**HM**	**CG**
**Repetition** (2 min)		Repetition of letter-sound couplingsand “*body phonemes*” for the simpleand conditional sounds for the letterS and E (practiced in session I).	Repetition of letter-sound couplingsand “*hand phonemes*” for the simpleand conditional sounds for the letterS and E (practiced in session I).	Repetition of letter-sound couplingsfor the simple and conditional soundsfor the letter S and E (practiced in session I).
**Activity I-IV** (12 min) Letter: L	See activity I-IV	Activity I-IV from session I is repeatedwith L/[l] in focus.	Activity I-IV from session I is repeatedwith L/[l] in focus.	Activity I-IV from session I is repeatedwith L/[l] in focus.
**Activity I-III** (7 min) Letter: M	See activity I-III	Activity I-III from session I is repeatedwith M/[m] in focus.	Activity I-III from session I is repeatedwith M/[m] in focus.	Activity I-III from session I is repeatedwith M/[m] in focus.
**Activity I-III** (7 min) Letter: Å	See activity I-III	Activity I-III from session I is repeatedwith Å/[ɔ] in focus.	Activity I-III from session I is repeatedwith Å/[ɔ] in focus.	Activity I-III from session I is repeatedwith Å/[ɔ] in focus.
**Activity VI** (7 min) Title: “*Lettersalat*” Letters: S, E, L, M, Å	Coupling of single letter sounds to small words	Standing in a circle each child placesfive sheets (A4) with the letters s, e, l,m and å in front of it on the floor.Words consisting of trained soundsare collectively deciphered onesound at the time. At identification ofa sound, the coupled body phonemeis performed simultaneously andsubsequently the child drags thesheet with the coupled lettertowards himself/herself. Afterdeciphering the word, the bodyphonemes and sounds areperformed in series multiple timeswith increasing speed to connect theindividual sounds and bodyphonemes into a word. Materials: Letter sheets (A4)	Sitting around a table each childplaces five small sheets (2 × 3 cm)with the letters s, e, l, m and å infront of it on the table. Wordsconsisting of trained sounds arecollectively deciphered one sound atthe time. At identification of a sound,the coupled hand phoneme isperformed simultaneously andsubsequently the child drags thesheet with the coupled lettertowards himself/herself. Afterdeciphering the word, the handphonemes and sounds areperformed in series multiple timeswith increasing speed to connect theindividual sounds and handphonemes into a word. Materials: Letter sheets (2 × 3 cm)	Children have a task on the controlsheet with five puzzle pieces labeledwith the the letters s, e, l, m and å.Below are pictures of wordsconsisting of trained sounds. Wordsare collectively deciphered one soundat the time. The child writes the lettercoupled to the sound below thepicture. After deciphering, the childreads the word aloud. Materials: Control sheet
**SESSION III**				
**Activity III**		**WM**	**HM**	**CG**
**Repetition** (5 min)		Repetition of letter-sound couplingsand “*body phonemes*” for the simpleand conditional sounds for the letterS, E, L, M and Å	Repetition of letter-sound couplingsand “*hand phonemes*” for the simpleand conditional sounds for the letterS, E, L, M and Å	Repetition of letter-sound couplingsfor the simple and conditional soundsfor the letter S, E, L, M and Å
**Activity VII** 12 min) Title: “*RhymeTwister*” Letters: s, e, l,m, å	Rhyming, letter sounds	In pairs, the children are facing afoam game board (1 × 1 m) with 16fields on each side. The 16 fieldscontain eight shuffled pairs of awritten word and a rhyming picture(e.g. the word *hair* and a picture of achair). The written words consist oftrained letters and sounds only. Onechild has the lead and reads aloudthe written words one by one. Afterreading a word, the leading childplaces a hand or a foot on the word.The other child now has to find therhyming picture matching the wordand place a hand or a foot on it. Thiscontinues until all words are read.The game board is turned aroundand the roles of the children areswitched. Materials: Rhyme Twister foam game boards(1 × 1 m) Sheets with words and pictures	In pairs, the children are facing agame board (12 × 12 cm) with 16fields on each side. The 16 fieldscontain eight shuffled pairs of awritten word and a rhyming picture(e.g. the word *hair* and a picture of achair). The written words consist oftrained letters and sounds only. Onechild has the lead and reads aloudthe written words one by one. Afterreading a word, the leading childplaces a thump or an index finger onthe word. The other child now has tofind the rhyming picture matchingthe word and place a thump or anindex finger on it. This continuesuntil all words are read. The gameboard is turned around and the rolesof the children are switched. Materials: Printed Rhyme Twister game boardswith words and pictures (12 × 12 cm)	In pairs, the children are facing agame board on control sheet A with16 fields. The 16 fields contain eightshuffled pairs of a written word and arhyming picture (e.g. the word *hair*and a picture of a chair). The writtenwords consist of trained letters andsounds only. One child has the leadand reads aloud the written wordsone by one. After reading a word, theleading child ticks the word. Theother child now has to find therhyming picture matching the wordand tick it. This continues until allwords are read. In control sheet B theroles of the children are switched. Materials: Control sheets (A and B)
**Activity VIII** (14 min) Title: “*Guess-a-word*” Letters: s, e, l,m, å	Coupling ofsingle lettersounds tosmall words	In pairs, the children are handed outfour cards each. On each card is aword consisting of trained letters andsounds only, and a picture illustratingthe word. The children take turn in“spelling” and guessing the words.The “spelling” child performs onebody phoneme and sound at a timeof the word on his/her card. Aftereach body phoneme, the other childrepeats the body phoneme andwrites the matching letter on ablackboard cloth. After the last bodyphoneme, the guessing child has toread the word on the blackboardcloth. Materials: Blackboard clothes and chalk “Guess-a-word” cards	In pairs, the children are handed outfour cards each. On each card is aword consisting of trained letters andsounds only, and a picture illustratingthe word. The children take turn in“spelling” and guessing the words.The “spelling” child performs onehand phoneme and sound at a timeof the word on his/her card. Aftereach hand phoneme, the other childrepeats the hand phoneme andwrites the matching letter on a smallblackboard. After the last handphoneme, the guessing child has toread the word on the blackboard. Materials: Blackboards and chalk “Guess-a-word” cards	In pairs, one child is handed outcontrol sheet A and the other childcontrol sheet B. They are not allowedto see each other’s sheets. On eachsheet are four words consisting oftrained letters and sounds only. Eachword is illustrated by a picture. Thechildren take turn in “spelling” andguessing the words. The “spelling”child says one sound at a time of theword on his/her sheet. After eachsound, the other child repeats thesound and writes the matching letteron his/her own sheet. After the lastsound, the guessing child has to readthe word on his/her sheet. Materials: Control sheets (A and B)

Protocol for 1 week (example: week 1)

*HM* hand movements, *WM* whole-body movements, *CG* control group

#### The story about the flying suitcase

To keep the children motivated and actively engaged a story about “*The flying suitcase*” will combine the activities. The flying suitcase, who loves fairytales, experiences something new every week (e.g. *circus*, farm etc.). The suitcase is the lead actor of the stories but also a physical item containing pictures of animals or things beginning with the letter-sounds in focus. In each of the first six weeks, the first two sessions starts with a small story about the flying suitcase and a short presentation of the pictures in the suitcase. In turn, the children get to pick up a picture and tell what they see on the picture.

### Content and organization of intervention- and control activities

#### Intervention conditions

The activities included in the two interventions vary mainly in regards to the motor modality used (i.e. hand vs. whole-body movements). In both interventions, activities have been developed with the embodied learning theory in mind, linking movement closely to the learning content. A basic element in the two interventions is the coupling of movements to letter sounds and letterforms. Children will be taught to perform a specific movement for every letter sound, and this movement-sound coupling will be used throughout the intervention. Short staccato-like letter sounds (e.g. the pronunciations of the letters “P”, “K”, “T”) are carried out as fast and powerful movements, whereas long letter sounds (e.g. the pronunciations of the letters “S”, “O”, “A”) are performed as slow and fluent movements. Moreover, when possible, the movement may be associated with objects or living creatures (e.g. the movement coupled to the sound of “S” may be associated with a snake). All movement-sound couplings are executed from left to right, i.e. the reading direction. Children in the WM group are encouraged to perform movements using their whole body and the space surrounding them. Activities in WM are mainly performed individually standing in a circle on the floor or in predefined couples/pairs. Activities in HM will be completed seated around a table and children are instructed only to use their arms and hands during the session. As for WM, activities in HM are performed individually or in predefined couples/pairs. Activities will be instructed by trained instructors and completed one at the time to ensure that all children understand and complete all tasks. Instructors will be asked to correct and support on a group level. Detailed descriptions of intervention activities are provided in Table 3.

#### Control condition

Control activities involve learning content similar to the activities in the two intervention groups and will be conducted in parallel with the intervention activities. Teachers are told not to encourage the children to use any motor movement (e.g. hand phonemes) besides handwriting during the control sessions. Importantly, though movements are reduced to a minimal, control activities still have a strong focus on letter sounds. All tasks will be completed seated on a chair, individually or in predefined couples/pairs, using paper and pencil. Before the intervention period, tasks are printed and handed out to the teachers who will administer the control activities according to standardized procedures (Table 3). As for the two interventions, each task will be handed out and completed one at the time to ensure that all children understand and complete all tasks. Detailed descriptions of control activities are provided in Table 3.

### Training of teachers and instructors

Before the intervention period, instructors and teachers will attend two separate training programs. At each school, teachers will complete a 2-h session in which a researcher will introduce the control material and provide the teachers with general guidelines of how to implement the activities. Instructors will complete two 3-h sessions comprising 1) introduction to the intervention activities and the target group and 2) practice of the intervention activities including correct pronunciation of each letter sound and the movement-sound couplings.

### Justification of sample size

The study has not been formally powered based on results from a specific study, as no equivalent study with similar outcomes exists. In general, the effects of physical activity lessons are observed to be small to moderate [60]. In addition, a study based on the embodied learning theory found small to moderate effect sizes in regards to letter knowledge when comparing learning activities involving whole-body movements with visual learning activities [4]. Given three groups with an equal number of participants in each group, power calculations suggest that 158 subjects are needed to achieve a power of 80% and a level of significance of 5% for detecting an effect size of 0.25 between groups. Since the control group will include approximately twice as many participants as each of the intervention groups, 212 subjects are needed to detect an effect size of 0.25 (intervention group: *n* = 53, control: *n* = 106). Accounting for a 20% dropout rate or data loss for other reasons, 265 participants will be included in the study. Power calculations have been done using Gpower [27].

### Blinding

Due to the nature of the interventions, instructors/teachers and participants will not be blinded to the intervention/control status during the intervention period. However, outcome assessors will be blinded to the intervention/control status of the subjects, except for measures of fidelity and motivation. Data analyst will be blinded to intervention/control status of the subjects.

### Outcome measurements

To evaluate the effect of the interventions, tests assessing reading related skills will be administered at the schools prior and post the 8-week intervention period by trained staff. In addition, a third data collection will be completed approximately three months after the cessation of the intervention period to evaluate potential retention effects. Primary outcomes assessing potential training effects include word reading (target words) and knowledge of trained letter-sound correspondences, while secondary outcomes assessing potential transfer effects include word reading (untrained words), letter fluency, general knowledge of letter-sound correspondences and word reading accuracy and speed. In addition, to explore potential mediators working memory, attention, intrinsic motivation, gross motor skills and fine motor skills will be assessed. At baseline, immediately post intervention and at three months follow-up, tests will be administered in groups and individually on two separate days. Compliance data will be collected following each session by the instructor/teacher and intrinsic motivation will be evaluated following four different sessions (week 2, 4, 6 and 8). All data will be collected during school hours which will ensure the children’s participation during data collection. In cases of absence on the day of scheduled data collection, children will be invited to complete the tests on another day.

#### Group tests

The group tests will be conducted in groups of 12–14 children in the classroom and will have a duration of approximately 60 min. During group testing, students will be placed with plenty of space between them to avoid copying.

##### Bogstaveprøve 2 [‘Letter test 2’]

This test has been widely used in the Danish school system. The test consists of two subtests assessing simple letter-sound knowledge and vowel identification, respectively. For the purpose of the present study, only the first subtest will be administered to assess standard letter-sound knowledge. The subtest will be administered strictly according to the manufacture’s description [43]. The subtest consists of 20 items (preceded by two practice items). Each item consists of a picture followed by five letters. The participants will be instructed to identify the first letter of the word illustrated by the picture. The word is read aloud by the instructor. Participants will be told to use a red pencil to mark the letters and use a blue pencil to mark if a mistake is made. The score is the number of items correct.

##### Ordlæseprøve 1 [‘Word reading test 1’]

As for letter test 2, this test is widely used in the Danish school system and will be administered strictly according to the manufacture’s description [42]. The test consists of 78 items (preceded by two practice items) and evaluates word reading accuracy as well as efficiency. In each item participants have to select one of four pictures that corresponds to a printed word. Words increase in length from two to four letters. Participants solve as many items as possible within a time limit of 4 min. Every minute children will be asked to change pencil color in order to monitor their progression throughout the test. The score is the number of correctly solved items (efficiency) and percentage of correctly solved items (accuracy).

##### Knowledge of trained letter-sound correspondences

To evaluate the children’s knowledge about letter-sound correspondences trained in the intervention and control period, a simple multiple-choice test has been constructed. The test consists of 15 trials. In each trial, a sound corresponding to a standard or a conditional pronunciation of a letter is said out loud by the assessor. Children are instructed to identify the correct letter matching the sound from a row of four letters. Before the test trials, children will be provided with one practice trial. The 15 sounds represent both standard and conditional pronunciations of the letters a, e, o, r, u and v (Table 2). Results are numbers of correct letter-sound correspondences.

##### Gross motor skills (flamingo balance test)

Gross motor skills including balance [9] has previously been linked to academic performance [10]. The flamingo balance test is a standardized test that assesses the ability to balance successfully on one leg [1]. The children will be asked to bend their free leg backwards and grasp the foot with their hand on the same side. Children are instructed to stand on one leg for 1 min. To become familiar with the test, children will be given one try before the actual test. The number of attempts needed to stand on one leg for 1 min will be counted for each leg. If a child put his/her foot down more than 15 times within the first 30 s of the test, the child will be excluded. Children will be provided with a badge showing their identification number. The test will then be recorded and analyzed afterwards. The test score is the sum of attempts with both legs; lower scores indicate better performance.

#### Individual tests

The individual tests will be conducted in a quiet room with one assessor and take maximum 35 min for each participant to complete. All tests will follow standardized procedures. Initially, the assessor will fill out a form with background information about, e.g. date of testing, sex, dominant hand, bilingualism etc.

##### Word reading with pictures (target words)

During the intervention period, all children will study certain words (*target words*) thoroughly. To evaluate the specific effect of the intervention on children’s ability to read the target words, a computer-based test has been constructed. This word reading test consist of 18 presentations of targets words; one target word per presentation. Below the target word are placed four pictures of which one illustrates the target word. For the children to give a correct answer, they will have to touch the picture matching the target word on the touch screen. They are asked to touch the correct picture as fast as possible. The three remaining pictures represents words (distractors) that have either the initial (two pictures) or the final (one picture) sound in common with the target word. The presentations of target words and the order of the four pictures are randomized between subjects and time points. During the test, children are allowed to ask questions concerning the pictures (e.g. *what is shown on this picture?*) but not regarding the target word (e.g. *what is the sound of this letter?*). The results from the test constitute mean response time for correct answers and number of correct answers.

##### Word reading without pictures (untrained words)

To test the potential transfer effects of the intervention on children’s ability to read small words, a computer-based test *not* including the target words has been constructed. This test consists of 12 presentations of untrained words; one word per presentation. The 12 words consist of four 2-letter words, four 3-letter words and four 4-letter words. Two versions of the test will be completed; one conducted at baseline and one at post intervention in a randomized and counterbalanced order. The two versions are matched carefully on letter sounds, numbers of letters and assumed difficulty. Pilot testing demonstrates no significant difference between performances in the two versions of the tests. Within each version of the test and within words with the same number of letters, words are presented in a randomized order. The child is instructed to read the word and say it aloud while looking at the assessor. Then the assessor registers the answer as correct or incorrect by pressing a green or red button, respectively. This procedure allows the child to read the word aloud for itself before providing an answer to the assessor. Each word will be presented for 16 s; providing the child with 15 s to get an answer registered (1 s is required for the assessor to register an answer). If no answer is given within the time limit, a new word will appear on the screen. If incorrect or no answer is provided for *all* four 2-letter words, the test is discontinued. The outcome is the number of correctly read words.

##### Naming of letter sounds (incl. The use of movement)

During the intervention there will be a strong focus on the coupling between letter sound and movement. To evaluate potential benefits of using movements when asked to remember letter sounds of a specific letter, a test has been constructed for the purpose. While standing, the child is asked to pronounce the letter sounds of specific letters (included letters are a, d, e, o, r, u and v which in Danish each have several possible pronunciations) which are read aloud by the assessor. The child will be told that he/she is allowed to use movements when pronouncing the letter sounds, but they are not directly encouraged to do so. For every letter sound, the child’s answer is registered as correct/incorrect/missing and whether movement/no movement was used. The results of the test are numbers of correct letter sounds pronounced in total, correct letter sounds pronounced using movements, and correct letter sounds pronounced without movements.

##### Letter naming fluency

A test of letter naming fluency will be included, since the speed of letter naming has been associated with e.g. word recognition and reading fluency [45, 47, 64, 76]. This Danish version of the Letter Naming test is based on the same principles as DIBELS Letter Naming Fluency [30] and has previously been used to evaluate children’s ability to name letters [65]. The test consists of a piece of paper (A4) with 12 rows of 10 letters (mixed upper- and lower case). The child is asked to name as many letters as possible in 1 min while pointing to each letter. Wrong namings are registered by the assessor. If the child does not name the letter within 3 sec, the assessor says the name of the letter and encourages the child to name the next letter. If all 10 letters in the first row are named incorrectly, the test is discontinued. One row of 10 letters will be provided as practice. Result from the test is numbers of letters named correctly.

##### Working memory

To our knowledge, there are no previous studies on pre reading, motor-enriched learning and working memory. Therefore, we include 1-back task as a visual working memory task and a digit span task as an auditory working memory task. Through these two working memory tasks, we can investigate covariation of unspecific working memory and the primary outcome measures of this study. Covariation of academic performance (in math) and working memory in longitudinal classroom-based interventions has previously been reported [5].

- **1-back task** To assess working memory a 1-back task will be completed by each child. A 1-back task was chosen, since a recent study including seven-year-old children reported a mean accuracy score between 60 and 70% in a comparable 1-back task, whereas the accuracy score in the included 2-back task was between 30 and 40% [63]. This 1-back task is constructed of representations of symbols (car, cloud, globe, note, headphones, airplane, plate, key, eye and bicycle); one symbol is presented at the time. On the 1-back task, children have to compare the symbol seen on the screen with the symbol previously presented. Children are instructed to press “yes” (a green key) on the keyboard if these two symbols are identical and “no” (a red key) if they are dissimilar, as fast and as accurate as possible. The test consists of 20 practice trials (30% “yes” trials) followed by two test blocks of 20 trials each (30% “yes” trials). Symbols are presented for 500 ms followed by a 3000 ms blank screen. The response window and inter-stimuli interval is 3500 ms each. The results from the test constitute number of correct and wrong “yes” and “no” answers, mean reaction time for correct “yes” and “no” answers, and number of non-responses in the two test blocks. Normative data on the n-back task for children and young adolescents have previously been reported [63].
- **Digit span** The digit span task is a subtest of the Wechsler Intelligence Scale for Children – fourth edition (WISC-IV) [88] and is used to assess auditory working memory, attention, and concentration. The test consists of two subtests; digit span forward and digit span backwards. In each subtest, participants hear a sequence of numerical digits after which they are asked to recall the digit sequence in the same order (subtest 1) or in reverse order (subtest 2). The sequences are said by the administer with a speed of one digit per second, starting with a sequence of two digits, then three digits, then four digits etc. Within each sequence length, two trials are provided. If the answer is incorrect on both these trials, the test is discontinued. The results from the tests constitute the number of correctly remembered sequences and the number of digits in the longest correctly remembered sequence.

##### Fine motor skills (9-hole pegboard)

The 9-hole pegboard test is a standardized test which has previously been used in children to evaluate fine motor skills [50, 75]. The 9-hole test consists of a square board with nine 1.3 cm (0.5 in.) deep holes which are spaced 3.2 cm (1.25 in.) apart. Sitting on a chair with the board placed in from of them, children are instructed to pick up the pegs one at a time, using the dominant hand only and put then into the holes in any order until the holes are all filled. Then the participant removes the pegs one at a time and return them to the container. The participant gets one practice trial before timing the test with a stop watch, starting when the child touches the first peg. The test is conducted with dominant hand and non-dominant hand. The test is conducted with the dominant hand first.

#### Motivation

A modified version of Intrinsic Motivation Inventory (IMI) [54] will be used to measure intrinsic motivation for the PLAYMORE activities. The original items of the IMI are translated into Danish using a translation-backtranslation process [77]. The inventory is reduced to six questions related to intrinsic motivation (question 1–3) and feeling of competences (question 4–6). Because the inventory will be used in children, the original 7-point likert scale is converted to a 4-point likert scale (1, not true at all, 2, only slightly true, 3, almost true; 4, true). Immediately after one session in week two, four, six and eight, an assessor will read the questions aloud for the children one by one. This inventory measures participants’ subjective experience related to an activity in an experiment and has previously been used in other studies on the motivational effect of integrating physical and learning activities in primary schools [86]. Means from question 1–3 and from question 4–6 will be calculated for each child and used as measures for intrinsic motivation and feeling of competences, respectively.

#### Compliance to the intervention

Teachers/instructors will be asked to keep a written log of attendance of each child. Specifically, data on 1) presence (yes/no), and 2) active participation (yes/no) will be registered. Moreover, teachers/instructors will note whether each session has been completed (yes/no) and to what degree it followed the protocol (scale 1–5).

### Plan for data analysis

In accordance with the Consolidated Standards of Reporting Trials (CONSORT) 2010 for randomized controlled trials [57], flow of participants will be reported. Descriptive statistics will be summarized across schools and classes for each group. Potential differences at baseline between subjects included and excluded in the analyses (e.g. drop-outs) as well as differences between groups at baseline will be tested. Further, compliance to the intervention will be summarized for each group. Intention-to-treat analyses will be performed to assess the effect of the intervention on primary as well as secondary outcomes. Multilevel mixed model will be used as this model is able to provide an unbiased estimate given missing data assuming data is missing at random conditional on variables included in the model [83]. Patterns of missing data will be investigated. To account for the cluster structure in the data (i.e. subjects within classes within schools), class and school will be included as random effects in the model. Due to the randomized design, which is used to ensure equal distribution of confounding variables between groups, we will only adjust for potential confounding variables (e.g. sex, age) which show unevenly distribution between groups at baseline. In addition, per protocol analyses will be conducted using above-mentioned approach including participants with a participation rate above 90%. In addition, structural equation models (SEMs) will be used for mediation analyses to explore potential mediators.

### Ethics and data security

The study is approved by The Faculty of Sciences Ethical Commitee at University of Copenhagen (#504–0032/18–5000). Before inclusion, all parents with children attending preschool at the included schools will receive written and oral information provided by the project leader. For a child to participate, written informed consent must be returned to the project leader by the parents/legal guardian prior to the baseline measurements (standard form S5, cf. https://www.nvk.dk/samtykkeerklaeringer). Participation in the study may be discontinued at all times with no obligation to provide a reason. To anonymize the data, each child will be provided with an identification number (ID number), and no participant identifying information will be stored alongside data. A transfer key coupling the ID number with personal information will be safely stored separately from the trial data in order to secure confidentiality. All rules from the Danish Data Protection Agency and General Data Protection Regulation (GDPR) will be followed during all phases of the PLAYMORE study. Analyses will be conducted by the project leader, who is not practically involved in the data collection or the completion of the intervention/control. In order to promote data quality, independent research assistants will complete double data entry. The results will be published in peer-reviewed journals and through presentations at international scientific conferences. Authorship will be in agreement with The Vancouver Recommendations.

## Discussion

The PLAYMORE study will provide additional insight into the effects of physical activity/movement on academic performance. Although, PA has previously been linked to reading performance, the evidence is inconclusive [3, 22, 51, 71] and only few experimental studies have previously investigated the effects of movement integrated into reading practice (i.e. based on principles of embodied learning) [4, 52]. Moreover, the potential influence of motor modality (e.g. hand movement vs. whole-body movements) is still unclear.

Within educational research concerning embodied learning, intervention studies are characterized by a wide variety of approaches differing in regards to e.g. motor modality, level of bodily engagement, task integration and congruency between movement and learning task [73]. Consequently, to enable a fair comparison between studies, thorough descriptions and characterizations of activities included in the different intervention studies are essential. Also, aspects that should be considered and discussed when characterizing embodied learning activities are the level of task integration, the level of bodily engagement [73] and congruency (i.e. congruency between the movement and the learning task) [40]. The two PLAYMORE interventions involve activities with a close temporal and meaningful coupling between the movements and the learning task, thus representing activities with a high level of task integration. However, the different activities (see Table 3) vary in regards to congruency between the movement and the learning task. Accordingly, activities range from “*Letter sound*” (i.e. activity II, Table 3) and “*Symbol-Sound Coupling*” (i.e. activity III, Table 3) representing a high level of congruency between movement (e.g. drawing the letter form/symbol while linking to a specific sound-movement coupling in which the movement and sound have similar characteristics) and learning task (i.e. coupling between letter form/symbol and sound) to “*Rhyme Twister*” (i.e. activity VII, Table 3) representing a low level of congruency between the movement (e.g. placing hand/feet on two rhyming words) and the learning task (i.e. rhyming, identifying similar sounds). Even though, it is recommended that congruency should be considered in the design of embodied learning interventions, the contribution of this factor to the effectivity of embodied learning activities is uncertain [73].

According to the taxonomy suggested by [73] the activities in the two PLAYMORE interventions may be characterized differently in regards to the level of bodily engagement, which may lead to different effects of the two interventions [73]. Skulmowski and Rey argue that a higher level bodily engagement may not automatically cause increases in learning performance (see [82] for a related discussion). However, in the embodied learning research, only few studies have previously compared the effects of part-body movements (e.g. hand movements) with effects of whole-body movements on subdomains of academic performance [5, 53], and neither of them focused on early reading skills in children. Thus, the inclusion of two intervention groups performing either part-body movements (i.e. arms and hands) or whole-body movements, respectively, will provide valuable information in regards to the influence of level of bodily engagement. Besides advancing our understanding of the relationship, this information will be of particular relevance to professionals implementing PA/movement into academic teaching. Compared to whole-body movements, hand movements may seem more practicable (e.g. requires little space, less time consuming) to the majority of teachers with no prior experience in organizing physical activities, and thus, if effective, hand movements may be more feasible to implement in the general school system.

The PLAYMORE study is unique as the methodology is stringent including theoretically founded activities that are well-described. Another strength of the study is the blinding of assessors at baseline and 8-weeks follow-up, thereby limiting the risk of bias. In addition, participants will be blinded at baseline, however, due to the nature of the study, it will not be possible to blind participants during the intervention period or at follow-up, which could introduce bias. However, with the age of the participants in mind, we do not expect this to influence the results significantly. The study includes a highly relevant control group completing the exact same academic content as the intervention groups, enabling a fair comparison between the groups. However, we are aware of the limitations within the control group and that these limitations may introduce bias. Usual teachers will administer the control activities while trained instructors will instruct the intervention activities, which may cause differences (e.g. comfort, newsworthiness) between the groups not associated with the differences in bodily movement. This decision is made for practical and ethical reasons, since it enables children without written consent to participate in the control activities along with their peers. In addition, the children-to-teacher rate is approximately twice as large in the control group compared to the intervention groups, which may affect the group dynamics. However, since teachers/instructors are asked to correct and support on a group level, the differences in children-to-teacher rate will most likely not affect the time spend correcting/supporting each child.

## Conclusion

The PLAYMORE intervention study will provide us with information about the immediate effect of the 8-week interventions and three months retention effects, but not whether potential effects transfer into better reading skills in the longer term. In its present forms, the two interventions contain components which may be directly incorporated into the teaching of beginner readers. However, if effective, evaluation of the implementation of the PLAYMORE program including more than six children in each group must be completed in order to investigate whether the program and the related effects can be transferred into standard school settings (e.g. numbers of children in each class, time and space restrictions). Lastly, if feasible, teacher training programs should be developed and implemented (e.g. into teacher education or further education) to enable an upscaling and potentially more widely use of the PLAYMORE activities in the Danish school system. So, as a final remark, the PLAYMORE study will lay the foundation for future research that have the potential to inform the political and scientific debate and importantly, to provide teachers with detailed information of how to implement PA effectively during teaching in order to support and motivate children in the process of learning to read.

## Data Availability

Not applicable.

## Abbreviations

PA: Physical activity
HM: Hand movements
WM: Whole-body movements
CG: Control group
